# Myocardial T1 and T2 Mapping in Duchenne muscular dystrophy: characterization of late gadolinium Eenhancement

**DOI:** 10.1186/1532-429X-18-S1-P279

**Published:** 2016-01-27

**Authors:** Jonathan H Soslow, Stephen M Damon, Kimberly Crum, David Parra, Andrew E Arai, Bruce M Damon, Larry W Markham

**Affiliations:** 1 grid.412807.80000000419369916Pediatrics, Division of Cardiology, Vanderbilt University Medical Center, Nashville, TN USA; 2 grid.412807.80000000419369916Department of Electrical Engineering and Computer Sciences, Vanderbilt University Medical Center, Nashville, TN USA; 3 grid.94365.3d0000000122975165National Heart, Lung, and Blood Institute, National Institutes of Health, Bethesda, MD USA; 4 grid.412807.80000000419369916Radiology and Radiological Sciences, Molecular Physiology and Biophysics, and Biomedical Engineering, Vanderbilt University Medical Center, Nashville, TN USA

## Background

In the current era, cardiovascular disease is the leading cause of death in patients with Duchenne muscular dystrophy (DMD). Pathological studies demonstrate myocardial fibrosis that begins in the subepicardium of the left ventricular free wall. Late gadolinium enhancement (LGE) images demonstrate a similar pattern of extracellular matrix (ECM) expansion. However, based on a baseline pro-inflammatory state and reports of possible myocarditis in DMD subjects, it is unclear whether this LGE represents edema or fibrosis. Our objective was to use extracellular volume (ECV) and T2 mapping to better characterize LGE in boys with DMD.

## Methods

21 DMD subjects and 11 healthy male controls were prospectively enrolled. Subjects underwent cardiac MRI including standard functional analysis and LGE as well as T1 and T2 mapping in the short axis at the level of the papillary muscles. Pre and post-contrast T1 maps and a hematocrit were used to calculate ECV maps. Manual contours were traced on ECV and T2 maps to determine global and segmental values. Focal regions of interest (ROI) in areas of significant LGE were traced on ECV maps. Values were compared between DMD and controls using a Wilcoxon rank-sum test.

## Results

**Figure 1:**
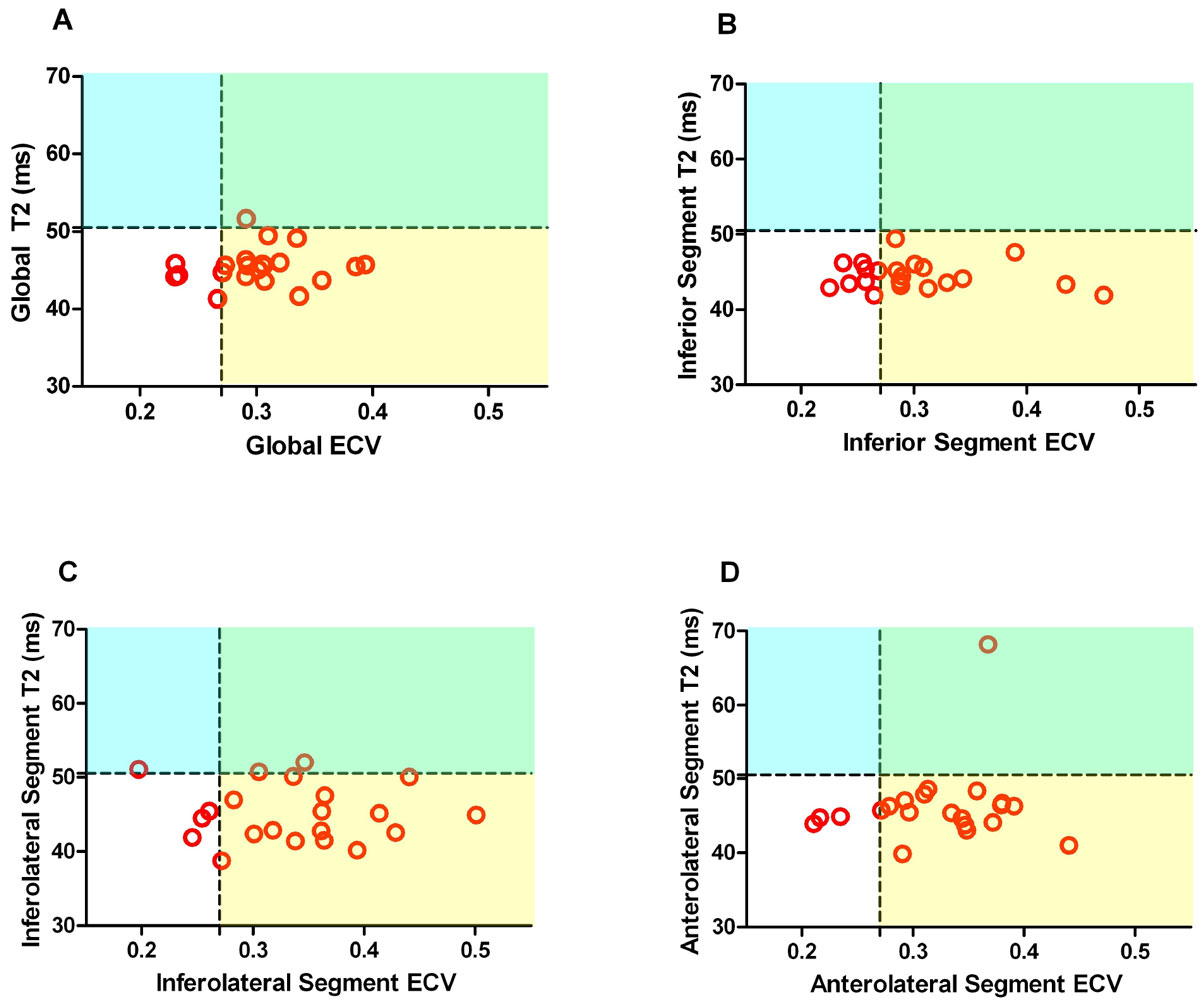
**Plot of T2 and extracellular volume (ECV) values for subjects with Duchenne muscular dystrophy**. A) Global T2 and ECV; B), C), and D)T2 and ECV of the inferior, inferolateral and anterolateral segments, respectively. Cut-offs are 2 standard deviations above the mean for control subjects. Values in yellow shading represent increased ECV and normal T2 suggestive of fibrosis, while values in blue represent increased T2 and normal ECV, suggestive of edema. Values in white represent "normal" myocardium without extracellular matrix expansion, while values in green either represent edema or a combination of edema and fibrosis.

DMD subjects were younger than controls (12.9 vs 25.0, p < 0.001) and had a similar LVEF (58.5% vs 60.3%, p = 0.347). 4 DMD subjects had LVEF < 55% and 12 had positive LGE. Global ECV was significantly elevated and global T2 was decreased in all DMD subjects compared with controls (0.30 vs 0.24, p < 0.001 and 45.5 ms vs 48.1 ms, p < 0.001). Normal cut-offs of 0.27 and 50.5 ms were derived for ECV and T2, both of which represent 2 standard deviations above the mean of control subjects. One DMD subject had global T2 greater than 50.5 ms (Figure 1A). Analysis of DMD segments with the highest incidence of LGE (inferior, inferolateral, and anterolateral) demonstrated ECV elevation in all three segments compared with controls (0.30 vs 0.22, 0.34 vs 0.24, 0.32 vs 0.24, p < 0.001 for all). T2 values in these segments were similar or decreased compared with controls (44.6 ms vs 46.5 ms p = 0.027, 45.1 ms vs 47.5 ms p = 0.061, and 46.3 ms vs 47.1 ms p = 0.081) (Figure 1B). The mean ECV in the focal ROIs with LGE was 0.48. Two DMD subjects had subjective areas of increased T2 in the same location as LGE, though these patches were smaller than the areas of LGE. One of these subjects had markedly elevated T2 in the corresponding segment (anterolateral = 68 ms). The remaining T2 maps were homogeneous.

## Conclusions

In this cohort, two DMD subjects had subjectively elevated T2 suggestive of focal inflammation or edema. The majority of DMD subjects had normal or decreased T2 and increased ECV in segments with LGE, suggesting that fibrosis is the primary etiology of ECM expansion.

